# Continuous Production of Methyl Lactate from Hemicellulosic Sugars: Identifying and Sorting out Sn-USY-Based Catalyst Deactivation

**DOI:** 10.1021/acssuschemeng.5c06986

**Published:** 2025-10-29

**Authors:** José Manuel Jiménez-Martín, Miriam El Tawil-Lucas, Ana Orozco-Saumell, Manuel López Granados, José Antonio Pulido, Rafael Mariscal, Jovita Moreno, Alicia García, Jose Iglesias

**Affiliations:** † Chemical & Environmental Engineering Group, Universidad Rey Juan Carlos, C/Tulipan s/n, Madrid 28933, Spain; ‡ Energy and Sustainable Chemistry (EQS) Group, Institute of Catalysis and Petrochemistry, CSIC, C/Marie Curie 2, Madrid 28049, Spain; § Instituto de Tecnologías Para la Sostenibilidad, Universidad Rey Juan Carlos, C/Tulipan s/n, Madrid 28933 Spain

**Keywords:** zeolite, methyl lactate, hemicellulose, continuous production, fixed bed reactor, deactivation

## Abstract

Potassium-exchanged tin-functionalized USY zeolite ([K]­Sn-USY) has been studied in the continuous transformation of glucose, xylose, and their mixtures in a fixed-bed reactor for the production of methyl lactate at 150 °C. The catalyst efficiently drives the transformation of all the studied substrates, though it faces several deactivation mechanisms, especially in the case of hexoses. Potassium leaching from the catalyst and organic deposition adduced to furanics produced during the reaction were ascribed as the major deactivation causes. The addition of small amounts (10 mg/kg) of potassium (as KCl or KOH) alleviated the catalyst deactivation, allowing the latter stable methyl lactate production over 30% yield for over 140 h from individual carbohydrates and complex sugar mixtures like Scots Pine hemicellulose hydrolysates.

## Graphical abstract

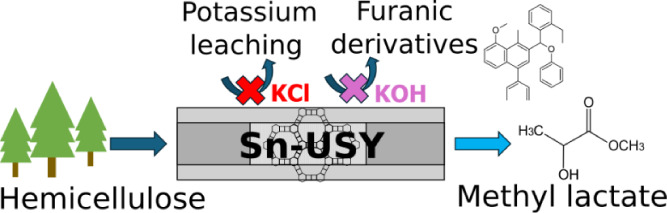

## Introduction

Alkyl lactates are highly interesting biomass derived chemicals finding application as green solvents, food additives, and synthons for the preparation of a wide variety of different chemicals.^1–4^ This makes alkyl lactates interesting platforms to prepare a wide variety of products that can play a key role in the transition to a biobased industry with reduced environmental footprints. The industrial production of lactic acid/methyl lactate involves a fermentative pathway using glucose as starting feedstock, which is accompanied by a high generation of waste (1 tm waste per 1 tm product), it is highly energy demanding (especially for product purification), and thus, there is plenty of room for improvement.^5^ Alternative pathways for the production of alkyl lactates have attracted attention in the last decades. In this regard, the use of glycerol^6^ and/or carbohydrates^7,8^ as substrates, both renewable biomass-derived raw materials, has attracted the attention of scientists in the past decade. Several strategies have been developed for their catalytic conversion,^5,9,10^ but from all the proposed alternatives, the transformation of carbohydrates in the presence of tin-catalysts has received most of the attention. Within this context, Sn-functionalized zeolites stand out as one of the most successful type of catalysts in the production of lactic acid derivatives from sugars, having demonstrated also a high activity and versatility in other Lewis acid driven transformations.^11,12^ Carbohydrates can undergo retro-aldol cleavage in the presence of tin-functionalized zeolites,^13^ producing small sugars like dihydroxyacetone (DHA), glyceraldehyde (GLY), or glycolaldehyde (GLA), which are excellent synthons for the production of a wide variety of hydroxy acids through a complex network of reactions, also catalyzed by Sn-functionalized zeolites (Scheme S1). Several factors affect the extension of the different transformations included in the complex reaction network taking place when treating carbohydrates with Sn-functionalized zeolites. Our own previous works on the use of these materials for the production of alkyl lactates have revealed the high intrinsic catalytic activity and selectivity of several of these zeolite structures in alkyl lactates synthesis.^14,15^ These catalytic systems can promote not only an efficient transformation of both hexoses, pentoses and their mixtures into alkyl lactates,^16^ but also bulky oligo- and poly saccharides^17^ if the textural properties are properly tuned. However, there is still a long way to go before considering the catalytic pathway as a serious alternative to fermentative lactic acid production processes. Some of the key challenges to face is developing an efficient continuous process to produce alkyl lactates for which robust and highly stable heterogeneous catalysts are required. These studies are scarce in the literature, and few examples have dealt with it. One of the first works was carried out by West et al. in 2010,^18^ transforming trioses into methyl lactate in the presence of a fixed bed of H-USY zeolite. Although the transformation was nearly quantitative, catalyst deactivation occurred after 48 h in water due to irreversible framework damage of the zeolitic structure. The more challenging transformation of glucose into methyl lactate with Sn-β was tested in continuous tubular reactors,^19,20^ yielding moderate yields of methyl lactate from diluted feed streams of glucose. Catalyst activity decline was ascribed to carbon deposition, framework damage, and Sn leaching. These phenomena were observed both in water and in methanol, though in the latter, the deactivation rate was less intense. Hammond and coworkers^21–24^ described similar deactivation phenomena for a Sn-β operating in a fixed bed reactor, though the addition of small amounts of water (<10 wt %) and potassium alkali salts to the reaction media partially alleviated it, demonstrating the system to be partially stabilized for some time (60 h). Despite these interesting findings, there is a lack of studies dealing with catalysts stability under long time-on-stream experiments. Moreover, the use of cellulose or hemicellulose hydrolysates, comprising complex mixtures of carbohydrates —which are closer to a real feedstock to be used on an industrial scale—, have been scarcely tackled in literature. Within this contribution, we present our advances on the transformation of sugar mixtures into methyl lactate, under continuous flow operation conditions, where deactivation phenomena effects are enhanced. Notably, several causes for catalyst deactivation phenomena have been identified, which seem to be linked and whose origin points to the leaching of potassium ions from the heterogeneous catalysts. Several remediation methods have been proposed, and the most efficient is applied to the treatment of complex carbohydrate mixtures, such as hydrolysates from Scots pine hemicellulose obtained through an organosolv fractionation method.

## Experimental Section

[K]­Sn-USY catalyst was prepared by postsynthetic modification of a commercial CBV-712 USY zeolite (SiO_2_/Al_2_O_3_ mole ratio of 12, Zeolyst). The parent material was calcined at 550 °C (room temperature to 200 °C at 1.8 °C/min; 360 min at 200 °C; 200 to 550 °C at 1.8 °C/min; 360 min at 550 °C) and dealuminated with aqueous nitric acid solution (10 M; 20 mL/g of zeolite) prior to metalation with SnCl_4_ in methylene chloride solution. After calcination, again at 550 °C, under the same conditions already mentioned (denoted as Sn-USY), the material was ion exchanged with a KCl aqueous solution (0.5 M) neutralizing Brønsted acidity. After drying at 110 °C, the recovered solid was calcined once again at 550 °C, using the same temperature profile, and denoted as [K]­Sn-USY.^15,16^

The catalytic performance of the prepared materials was evaluated in a homemade fixed-bed reactor consisting of a stainless steel tube of 1/4″ OD with upstream flow. The reactor was heated with a clamshell furnace at 150 °C and controlled with a PID controller (see Figure S1). The system was pressurized with 100 N mL of nitrogen at 13 bar. Typically, 0.5 g of the catalyst was loaded in the middle of the reactor tube between two glass wool layers, with glass beads (Sigma-Aldrich) used to fill the rest of the tube. The feed consisted of a solution of different saccharide substrates in methanol:water (96:4) media. The feed rate was stablized at 0.05 mL·min^–1^ for all the studied substrates (WHSV = 0.24 g_glucose_·g_cat_^–1^·h^–1^; 0.24 g_xylose_·g_cat_^–1^·h^–1^; 0.24 g_DHA_·g_cat_^–1^·h^–1^; 0.08 g_GLA_·g_cat_^–1^·h^–1^; 0.24 g_hemicellulose sugar_·g_cat_^–1^·h^–1^). The concentrations of the methanolic solutions used as feedstream were: [glucose] = 40 g·L^–1^; [xylose] = 40 g·L^–1^; [DHA] = 40 g·L^–1^; [GLA] = 13.3 g·L^–1^; [emulated scots pine hemicellulose] = 40 g·L^–1^; [real scots pine hemicellulose] = 40 g·L^–1^. The outstream of the reactor was collected in a coselector pressurized at 13 bar with 100 N mL N_2_ flow. Samples were withdrawn from the collector at specific times. Time zero was considered after the reactor reached 150 °C and the corresponding residence time. Collected samples were filtered with 0.2 μm filters prior to analysis. The *in situ* calcination of the catalyst was carried out in the same tubular reactor after displacement of reaction media with methanol and dried for 10 min with 100 N mL·min^–1^ of N_2_. After drying the catalyst, the gas feeding was changed to 100 N mL·min^–1^ of air, and the system was heated to 550 °C for 6 h. The cooling of the calcined catalyst fixed bed was conducted under air flow.

Spent catalyst has been analyzed by means of thermogravimetric analysis (TGA), elemental analysis, and FTIR. TGA has been conducted with a Mettler-Toledo TGA DSC-1, with 100 mL·min^–1^ air flow and 5 °C·min^–1^ slope, with ≈12 mg of sample loaded to the crucible. Elemental analysis was performed with a ThermoScientific Flash 2000 Organic Elemental Analyzer, with an operating temperature of 950 °C in the oven, and loading ≈2 mg of sample in tin crucibles. FTIR spectra were collected with a ThermoScientific Nicolet iS50 FT-IR in the range 4000–400 cm^–1^, with 32 scans.

Collected samples from the catalytic test were analyzed by means of HPLC and GC. HPLC analysis of sugars and methyl glycosides was performed with an Agilent 1260 unit using a Shodex Asahipack NH2P-50 4E column operating at 30 °C with acetonitrile/water (80:20; isocratic) as the mobile phase (1 mL·min^–1^). HPLC was connected to an Agilent 1260 Infinity ELSD detector, which was used for the quantification of sugars and glycosides. Quantification of reaction products was carried out with a Varian CP3900 GC unit, fitted with a CP WAX 52-CB column and an FID detector (operating at 230 °C), using helium as the carrier gas (oven temperature program: start at 50 °C, ramp 20 °C·min^–1^ up to 100 °C; ramp 40 °C·min^–1^ up to 140 °C; ramp 10 °C·min^–1^ up to 170 °C; ramp 40 °C·min^–1^ up to 230 °C; holding for 5 min). For quantification, *n*-decane was used as an internal standard. The quantification of sugars and methyl glycosides using pentoses as substrates was carried out by means of GC analysis with prior derivatization of the samples, following a methodology based on the procedure described by Pienkoß et al.^25^ Substrate conversion (*X_i_*), product yields (*Y_i_*), and product selectivity (*S_i_*) were calculated as follows:

1 $$X_{i}\left(\%\right)=\frac{\mathrm{Reacted}\;\mathrm{moles}\;\mathrm{of}\;\mathrm{substrate}}{\mathrm{Initial}\;\mathrm{moles}\;\mathrm{of}\;\mathrm{substrate}}\times 100$$

2 $$Y_{i}\left(\%\right)=\frac{\mathrm{Number}\;\mathrm{of}\;\mathrm{moles}\;\mathrm{of}\;\mathrm{product}\;i}{\mathrm{Initial}\;\mathrm{moles}\;\mathrm{of}\;\mathrm{substrate}\;i\times \mathrm{stoichiometric}\;\mathrm{coefficient}}\times 100$$

3 $$S_{i}\left(\%\right)=\frac{\mathrm{Number}\;\mathrm{of}\;\mathrm{moles}\;\mathrm{of}\;\mathrm{product}\;i\times \mathrm{stoichiometric}\;\mathrm{coefficient}}{\underset{i}{\sum }\left(\mathrm{Number}\;\mathrm{of}\;\mathrm{moles}\;\mathrm{of}\;\mathrm{product}\;i\times \mathrm{stoichiometric}\;\mathrm{coefficient}\right)}\times 100$$

The stoichiometric coefficient refers to the number of carbon atoms of the starting substrate divided by the number of carbon atoms in the considered product.

## Results and Discussion

### Transformation of Monosaccharides

Hemicellulose obtained from lignocellulosic biomass is a matrix of polysaccharides composed of different carbohydrate complexes, such as xylans, mannans, and xyloglucans. Hemicellulose hydrolysates contain hexoses and pentoses, including glucose, mannose, galactose, arabinose, and xylose, though glucose and xylose are the main components in most of the plants. Both monosaccharides were selected as substrates to test the ability of [K]­Sn-USY to drive their transformation to alkyl lactates.

#### Glucose

Glucose has been selected as a hexose representative for the evaluation of the catalytic performance of a K-exchanged Sn-USY-based catalyst ([K]­Sn-USY) loaded in a fixed-bed reactor, operated at 150 °C, 0.05 mL^–1^ feed rate, and 40 g·L^–1^ of glucose. Those conditions were selected based on batch condition optimization and a kinetic study of hexose transformation.^16,26^ Figure 1A depicts the yield and product distribution obtained after 160 h of continuous operation. [K]­Sn-USY exhibits high catalytic performance during the first hours, featuring a product distribution with methyl lactate (MLA) as the main product, accounting for a total 47% yield, together with other α-hydroxyesters like methyl vinyl glycolate (MVG), methyl 2-hydroxy-4-methoxybutanoate (MMHB), and a minor presence of glycolaldehyde dimethyl acetal (GADMA) and methyl glycolate (MG) with a total combined yield of 68%. It is also noteworthy that there is a high proportion of C4 chemicals which are derived from either the retro-aldol cleavage of glucose—yielding a tetrose and glycolaldehyde—or from the aldol condensation of the latter (Scheme S1). Other products include methyl glycosides, which are formed with low extension during the early stages of the test. Glycosidation of the alcohol solvent is driven by Brønsted acid sites, which are partially neutralized in [K]­Sn-USY by ion exchange. In a similar way, products derived from hydrolytic sugar transformations, such as 5-hydroxymethyl furfural (HMF) and methoxymethyl furfural (MMF), which are also catalyzed by Brønsted acid sites, are barely detected.

**Figure 1:**
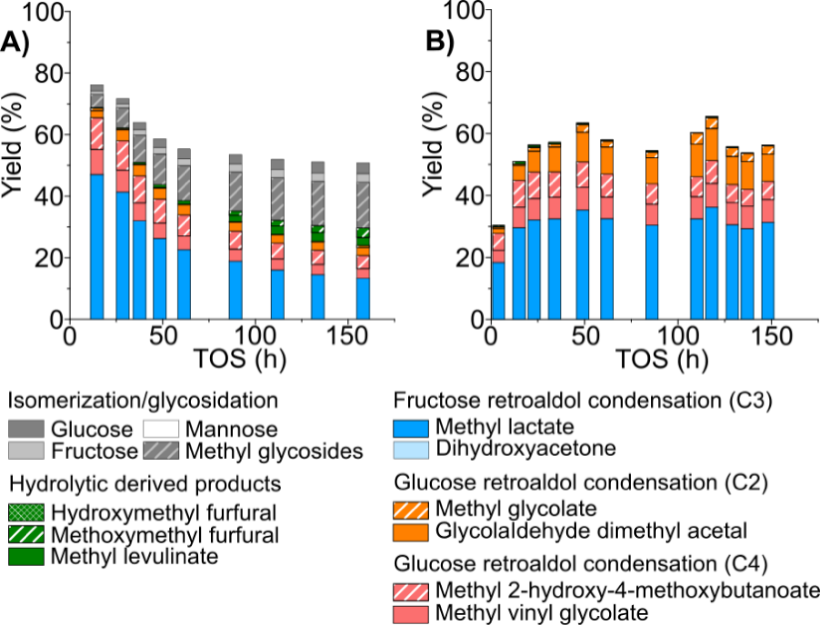
Product distribution obtained in the continuous transformation of glucose (A) and xylose (B) with the [K]­Sn-USY catalyst. Conditions: catalyst loading = 0.5 g; reaction medium = methanol:water (96:4 wt:wt); feed rate = 0.05 mL·min^–1^; WHSV = 0.24 g_sugar_·g_cat_^–1^·h^–1^; reaction temperature = 150 °C; pressure = 13 bar (N_2_ pressurized); N_2_ flow rate = 100 N mL·min^–1^.

Although [K]­Sn-USY has depicted quite a good reusability under batch reaction conditions,^16^ a clear deactivation of the catalyst is evident in the test performed in the fixed-bed reactor, taking place more intensely during the early stages of the reaction. Along with the decrease in the yield toward retro-aldol-derived products, the formation of glycosides and HMF progressively increased until the end of the test, pointing to a deactivation of Lewis acid sites (Sn centers), which are responsible for the retro-aldol cleavage of sugars. An attempt to reactivate the catalyst was carried out by *in situ* catalyst calcination in air flow. This strategy led to higher yields of retro-aldol cleavage-derived products, but only for a few hours (see Figure S2). On the other hand, the formation of glycosides and HMF remained at high levels, and the yield of these products permanently increased after 200 h of operation. These results, both before and after catalyst calcination, point to two different deactivation contributions: the formation of organic deposits onto the Lewis Sn sites, thus blocking them, and to an increase in the strength and/or population of Brønsted acid sites. The fact that the calcination treatment partially recovered the initial catalytic activity supports the formation of organic deposits on the catalyst. However, the increased production of HMF and related products suggests an increase of Brønsted acidity during operation, which might be related to the leaching of potassium ions. The preparation of [K]­Sn-USY involves an ion exchange step in which the zeolite is contacted with an aqueous solution of potassium chloride to reduce the Brønsted acidity associated with remaining framework aluminum species in the zeolite matrix.^27^ The efficient acidity reduction achieved through this simple pathway is evidenced by Py FTIR (Figure S3). The increase of Lewis:Brønsted acid ratio plays an important role in the modulation of the catalyst selectivity toward retro-aldol transformations, reducing the formation of products from other chemical pathways. Moreover, the presence of potassium also enhances the catalytic activity of Sn sites for retro-aldol condensation pathways,^15,28^ so their loss should also be accompanied by a drop in the catalytic activity. The tested reaction conditions, with the presence of water in the feed stream, might lead to progressive potassium leaching and to an increase in the number of Brønsted acid sites, shifting the selectivity of the zeolite to side products that cause the deactivation of the catalyst. Indeed, the formation of induced Brønsted acidity due to water adsorption over Sn Lewis acid centers has been demonstrated by Ivanova et al.^29^ In this way, even if potassium is not leached, the presence of water might be related to the promotion of those products causing deactivation. ICP-OES analysis of the product stream reflected a negligible potassium content (below the quantification limit). However, the spent catalyst was analyzed by ICP-OES with a similar procedure to that applied to fresh samples (see Experimental Section), evidencing that a loss of around 75% of potassium loading (decreasing from 0.4 wt % to 0.1 wt %) occurred after 440 h of time-on-stream, supporting our hypotheses.

#### Xylose

Pentoses are the most abundant monosaccharides in the majority of hemicelluloses, with xylose being the main contributor in most of vegetable species.^30^ For this reason, xylose has been used as a substrate to evaluate the possibility to prepare methyl lactate from hemicellulose with a [K]­Sn-USY catalyst under the same conditions used for glucose. Experiments conducted under batch reaction conditions^16^ reflected a good catalytic performance in the transformation of xylose and arabinose. The results obtained in the fixed-bed reactor under continuous flow conditions are presented in Figure 1B and reflect a product distribution featuring methyl lactate as the main product, similar to glucose, though yielding 32%, along with C2- and C4-derived products, with GADMA, MVG, and MMHB presenting yields of 8%, 7%, and 8%, respectively. These results are quite similar to those achieved under batch conditions. On the other hand, no unconverted xylose, its isomers, or methyl pentosides were formed in quantities enough to be detected. Interestingly, deactivation phenomena were less abrupt when treating xylose compared to the test with glucose. No great differences in product distribution were detected along the catalytic experiment, with the most important one being the slight increase in the yield toward GADMA observed during the first 50 h. This might suggest some loss of catalytic activity in aldol condensation reactions, but the observed differences are too small to be conclusive. On the other hand, despite the extent of potassium leaching being similar to that observed when treating glucose, the main difference when comparing the spent catalysts after treating glucose or xylose lies in the formation of organic deposits on the surface of the catalysts, which were higher in the case of hexoses.

#### Dihydroxyacetone and Glycolaldehyde

The formation of the organic deposits on the surface of the catalysts seems to be related to the existence of side reactions, which could be ascribed to the condensation of highly reactive reaction intermediates to bulky products finally deposited on the catalytic sites. Aiming to understand such a role, the most abundant reaction intermediates found in the catalytic tests —dihydroxyacetone (DHA) and glyceraldehyde (GLA), in concentrations equivalent to that obtained in the transformation of hexoses and pentoses, respectively—both produced in the conversion of hexoses and pentoses, were tested as reaction substrates to evaluate their ability to cause the deactivation of the catalyst.

The assay conducted with DHA (Figure 2A) reflects the good catalytic performance of the catalyst in the isomerization of the substrate to lactate moieties, showing high conversion and selectivity toward the target product, with an average yield of 70% in the first 100 h and 100% selectivity. These results are quite similar to those achieved under batch reaction conditions,^16^ though substrate conversion was not complete in the tests reported here due to a lower contact time between the catalyst and the reaction media. More interestingly, no great decrease in the catalytic activity of the [K]­Sn-USY zeolite was observed for over 265 h of time on stream, with a high material balance, which was well kept during the operation run, which evidence that DHA is not related to any of the causes of catalyst deactivation occurring when treating sugars as substrates.

**Figure 2:**
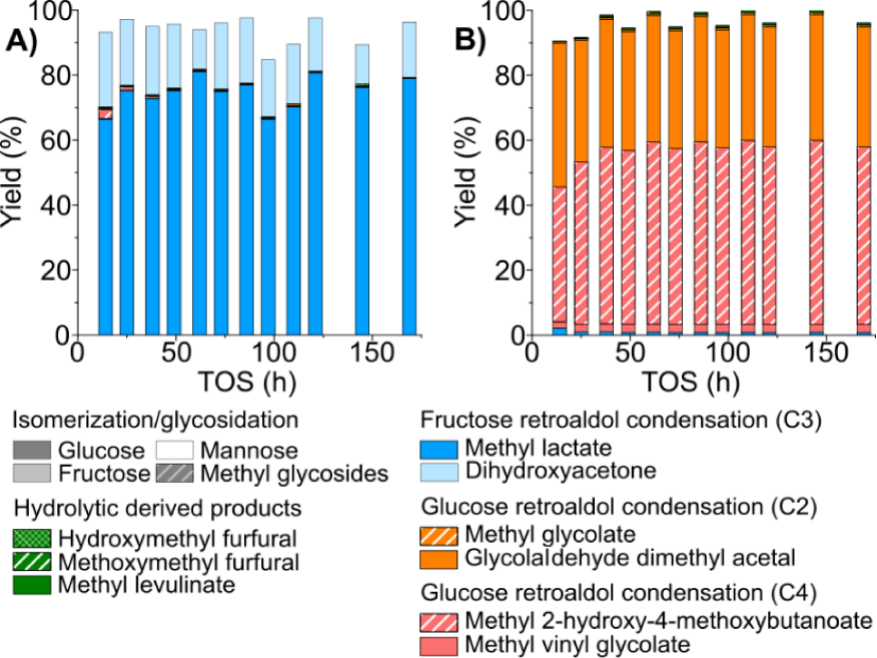
Product distribution obtained in the continuous transformation of (A) DHA and (B) GLA using [K]­Sn-USY. Catalyst loading = 0.5 g; reaction medium = methanol:water (96:4 wt:wt); feed rate = 0.05 mL·min^–1^; reaction temperature = 150 °C; pressure = 13 bar (N_2_ pressurized); N_2_ flow rate = 100 N mL·min^–1^. Specific conditions: (A) DHA conc. = 40 g·L^–1^; WHSV = 0.24 g_DHA_·g_cat_^–1^·h^–1^; (B) GLA conc. = 13.3 g·L^–1^; WHSV = 0.08 g_GLA_·g_cat_^–1^·h^–1^.

Having not detected any catalyst deactivation, the feed stream of the reactor was switched to a methanolic GLA solution, using the same catalyst bed and taking the first sample after the total volume of the reactor was displaced. Similarly to that observed with DHA, the conversion of GLA was very high since the beginning of the operation, well above 90% (Figure 2B). Two main products were detected, MMHB and GADMA, being steadily produced for 150 h with yields of 60% and 38%, respectively. This product distribution agrees with our previous studies, in which glycolaldehyde is either acetylated with the methanol solvent, producing GADMA, or condensed into a tetrose forming the C4 carbon backbone of MMHB. In this regard, it is interesting to note the distribution of C4 products, with the major contributor to this group being MMHB, accompanied by very low amounts of MVG. This result contrasts with those achieved for the same catalyst in a batch reactor,^15^ as the total yield of C4 products was distributed in similar amounts between MMHB and MVG. These differences can be ascribed to the distinct contact times between the catalyst and the reaction media in both types of reactors—6 h in the batch reactor vs <25 min in the fixed-bed reactor—which depresses the extension of the more demanding dehydration (conducting to MVG) as compared to the etherification with methanol (leading to MMHB). Regarding the material balance for the GLA transformation, it remains above 95% during all the operation run, lasting for 150 h without exhibiting any deactivation or change in product distribution. This test, together with that previously carried out with DHA, accounted for a combined total time of 400 h of continuous operation without any signs of deactivation of the catalyst. The catalytic performance exhibited by [K]­Sn-USY in the transformation of both DHA and GLA highlights its versatility and effectiveness in valorizing different carbohydrates, making it suitable for targeting different bioproducts within a biorefinery scheme. Additionally, the outstanding stability observed in the transformation of DHA and GLA evidence that these intermediates of the retro-aldol splitting of sugars are not related to the deactivation of the catalysts when treating monosaccharides. In this way, other side products, such as methyl glycosides, produced by the etherification of the substrates with the alcohol solvent, or furanics, evolving from hydrolytic pathways undergoing the sugar substrates, must be related to the formation of the organic deposits blocking the catalyst sites. It is noteworthy that both side products—methyl glycosides and furanics—are produced in Brønsted acid sites, so the leaching of potassium ions, the formation of side products, and the catalyst deactivation could be intimately related.

To explore such a possibility, experiments in batch conditions were conducted using the same catalyst and temperature conditions but treating methyl glucoside and 5-HMF as the starting substrates. The transformation of methyl glucoside presented a complete lack of activity, with negligible methyl lactate formation and negligible deposition on the catalyst (as evidenced by TGA). However, the transformation of 5-HMF led to the formation of methoxymethyl furfural, methyl levulinate, and polycyclic organic compounds, detected by GC-MS analysis of the reaction media. This finding is an indication of the potential of furanics to form heavy compounds inside the pores of the zeolite, causing pore blocking and consequent catalyst deactivation. Small amounts of free 5-HMF in the reaction medium confirmed its high reactivity under the tested conditions and the affinity of the furanics for the catalyst surface. However, these organic deposits can be easily removed by calcination, so that eventually the catalytic activity of the [K]­Sn-USY zeolite can be recovered by a simple thermal treatment.

### Characterization of Spent Catalysts

Spent catalyst after transformation of glucose and xylose was analyzed by means of TGA, FTIR, and elemental analysis to further assess the deactivation mechanism. Thermogravimetric analysis of the spent catalyst (Figure S4) depicts the mass loss and mass loss derivative of the catalyst after transformation of glucose (A) and xylose (B). Attending to the total mass loss, the catalyst used in the transformation of glucose presents a 13.3% mass loss, while in the case of xylose, the observed mass loss is 9%. The mass loss derivative, presented in blue, indicates that glucose exhibits the main mass loss process after 300 °C, while xylose presents the mass loss events between 200 and 300 °C, suggesting heavier deposits in the case of glucose. This is aligned with the higher catalytic stability observed for [K]­Sn-USY with these substrates. Elemental analysis (see Table S3) also indicates a higher carbon content in the case of glucose, reinforcing the hypothesis of higher organic deposition on the catalyst. FTIR spectra of spent catalysts after the transformation of glucose and xylose (Figure S5) present vibration signals located at 1730 and 1756 cm^–1^, signals that can be attributed to C=C in furanic compounds. The signal located at 2930 cm^–1^, which is also present in the samples after the reaction, can be ascribed to C–H bond vibrations. The presence of those signals on the spent catalysts suggests the presence of furanic and/or furanic polycondensed compounds, playing an important role in the proposed deactivation mechanism.

### Remediation of Catalyst Deactivation

Based on the catalytic performance exhibited under continuous flow conditions with hexoses, pentoses, and reaction intermediates, two interconnected deactivation mechanisms are proposed: a) loss of intrinsic catalytic activity and furanic compounds produced due to the increased population of Brønsted acid sites, related to potassium leaching and/or induced Brønsted acidity in the presence of water and b) deposition of organic deposits derived from condensation of those furanics.

As an increase in the number of Brønsted acid sites is involved in both the identified deactivation causes, directly or indirectly, and the link with evidenced potassium leaching, this is the first issue to be tackled. Potassium is a fundamental additive of Sn-functionalized zeolites to enhance the selectivity in the production of methyl lactate from sugars. These not only promote the activity by interacting with the tin sites, but they also neutralize residual Brønsted acidity from the parent zeolite by ion exchanging the acid protons associated with remaining framework aluminum sites and with water molecules interacting with water. Compensating potassium lixiviation by the addition of small traces of potassium salts to the reaction media could be an interesting option to minimize the regeneration of Brønsted acidity and the consequent formation of side products, which finally leads to catalyst deactivation.

#### Addition of KCl

The addition of potassium chloride ([KCl] = 5 mg/kg) to the reaction medium has been used to minimize potassium leaching. Padovan et al.^22^ already demonstrated the benefits of the addition of potassium salts to improve the catalytic stability of a Sn-β catalyst in the isomerization and retro-aldol transformation of glucose. We applied the same strategy in the case of [K]­Sn-USY through the addition of potassium to compensate for potassium leaching. Previously, KCl was demonstrated to be inactive in the transformation of sugars to methyl lactate under the tested reaction conditions. In this way, the influence of its presence in the reaction media, as presented below, corresponds solely to the preservation of the catalytic activity of [K]­Sn-USY.

The addition of KCl to the feed stream does not prevent the deactivation of [K]­Sn-USY in the transformation of glucose to methyl lactate, as is clear in Figure 3. However, compared with the catalytic assay conducted without the addition of KCl to the reaction media (Figure 1A), several important differences are detected. First, the catalyst deactivation rate seems to be slower compared to the test performed in the absence of KCl. Second, products coming from hydrolytic routes, such as HMF or related chemicals (methoxy methyl furan, methyl levulinate), are absent among those detected in the reaction media when using KCl. This means that the Brønsted acidity of the catalyst was not easily regenerated if K^+^ ions are present in the reaction media, and thus, the chemical routes that Brønsted acidity promotes are prevented. After the first operation run, a calcination treatment to remove organic deposits and regenerate the catalyst was applied. This treatment seems to be effective, as it allows the recovery of the initial catalyst activity without observable changes in product distribution. However, the evolution of the product distribution was similar, and catalytic activity dropped to values similar to those obtained after long time-on-stream values in the first assay. After a second calcination treatment applied to the very same catalyst, the concentration of potassium in the reaction media was increased to 10 mg/kg, aiming to boost its beneficial influence. No relevant changes in the catalytic activity were observed, recovering the starting catalytic activity, but the catalyst undergoes a similar deactivation, though at a slower rate. After three runs, a total time on stream period of more than 400 h was completed, describing the same product distribution profile and catalytic performance. This behavior evidence that the addition of KCl to the reaction media slightly delays the deactivation of the [K]­Sn-USY catalyst, allowing its use in a continuous fixed-bed reactor with easy regeneration by *in situ* calcination. Nevertheless, KCl does not prevent the deactivation of the catalyst, most probably because potassium ions just exchange the acid protons created on the tin sites, but Brønsted acidity is not neutralized, and thus, the reactions it promotes are still taking place.

**Figure 3:**
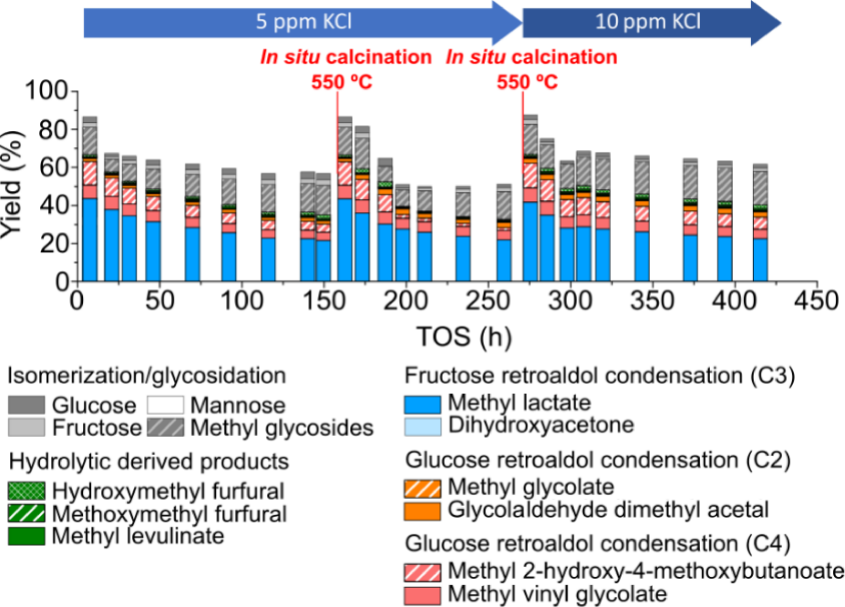
Product distribution obtained in the continuous transformation of glucose with the [K]­Sn-USY catalyst at different concentrations of KCl in the feed stream. Conditions: catalyst loading = 0.5 g; reaction medium = methanol:water (96:4 wt:wt); glucose concentration = 40 g·L^–1^; feed rate = 0.05 mL·min^–1^; WHSV = 0.24 g_sugar_·g_cat_^–1^·h^–1^; reaction temperature = 150 °C; pressure = 13 bar (N_2_ pressurized); N_2_ flow rate = 100 N mL·min^–1^.

#### Addition of KOH

The addition of KOH as an alternative to KCl has been tested as an additive to compensate for the leaching of potassium ions. However, together with this effect, the purpose of moving to KOH is also to evaluate its capability to neutralize the induced Brønsted acidity. This double effect is expected to preserve the activity of the catalyst in sugar retro-aldol cleavage while reducing the formation of induced acid sites, thus preventing the formation of deactivating furanics. Figure 4 presents the catalytic tests conducted with the addition of 10 mg/kg of KOH to the reaction media.

**Figure 4:**
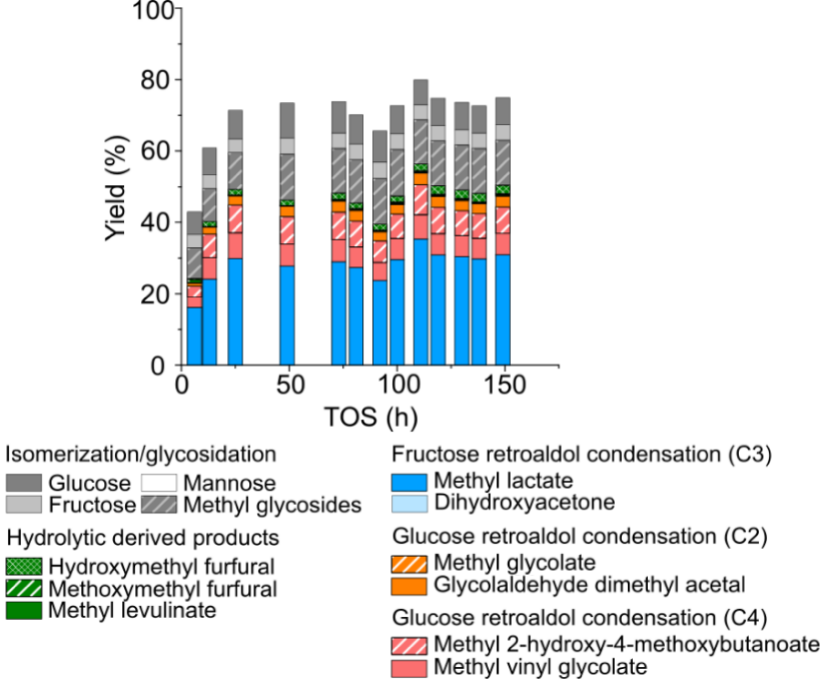
Product distribution obtained in the continuous transformation of glucose with the [K]­Sn-USY catalyst with 10 ppm of potassium hydroxide in the feed stream. Conditions: catalyst loading = 0.5 g; reaction medium = methanol:water (96:4 wt:wt); glucose concentration = 40 g·L^–1^; WHSV = 0.24 g_sugar_·g_cat_^–1^·h^–1^; reaction temperature = 150 °C; pressure = 13 bar (N_2_ pressurized); N_2_ flow rate = 100 N mL·min^–1^.

After 24 h of progressively increasing product yields, a stable operation regime is achieved, featuring a methyl lactate yield of 30%, which is well preserved after 150 h on stream. This result evidenced the beneficial effect of KOH in preserving the activity of the [K]­Sn-USY catalyst in the production of methyl lactate. Alongside methyl lactate, the product distribution does not present great differences with the benchmark experiment—conducted in the absence of KOH—yielding MMHB and MVG with a 7% yield each and showing no changes in product distribution during the overall operation. The presence of side products derived from hydrolytic reaction pathways—promoted by Brønsted acid sites—was detected at low concentration, being limited to the production of methoxymethyl furfural and methyl levulinate. On the contrary, the mass balance of the transformation was improved by 15%, which could be ascribed to the reduction of organic deposits, presumably products derived from the condensation of furanics. In addition, the formation of methyl glycosides—another Brønsted acid-promoted reaction—was also reduced, which was accompanied by an evident increase of the concentration of free monosaccharides such as fructose and glucose. Based on the effects and changes observed in the catalytic performance of [K]­Sn-USY in the presence of small amounts of KOH, our results point to a neutralization of the Brønsted acid sites in the catalysts, which are responsible for the formation of side products conducting to the deactivating organic deposits detected in the spent catalysts. By preventing these side reactions, the catalyst shows a stable performance that allows the production of methyl lactate from glucose without regeneration, lasting for over 140 h.

To evaluate the influence of the addition of potassium additives on the acidity of the [K]­Sn-USY zeolite, several DRIFT analyses, using deuterated acetonitrile as a molecular probe, were conducted for catalyst samples exposed to three different media (methanol:water (96:4); methanol:water (96:4) + 10 mg/kg KCl; methanol:water (96:4) + 10 mg/kg KOH). Figure 5 depicts the DRIFT spectra collected for [K]­Sn-USY samples exposed to a mixture of methanol and water and the potassium additives used in our tests. The spectrum collected for the sample contacted with the solvents but in the absence of potassium salts presents contributions attributed to strong Brønsted acid sites (2298 cm^–1^, ascribed to those in remaining framework aluminum sites and/or those formed by water dissociative adsorption on tin sites), weak Brønsted acid sites (2271 cm^–1^ ascribed to silanol groups), and Lewis centers (2309 and 2316 cm^–1^, corresponding to closed and open Sn sites, respectively). Regarding tin centers, the most abundant species are closed sites. After exposing the material to the same mixture, but with KCl, a general weakening of the adsorption intensity is observed, indicating that the presence of potassium in the medium is exerting a reduction of the acidity of tin sites or at least on its detection. The reduction of acidity associated with Al and Sn sites suggests the addition of potassium might neutralize acidity in the heterogeneous catalyst by ion exchange in Brønsted acid sites and by interaction with metal centers in Lewis acid sites. This latter most probably causes the opening of closed tin sites observed for the sample treated with KCl. When KOH is present, the [K]­Sn-USY catalyst exhibits nearly no signals in the region ascribed to deuterated acetonitrile adsorbed onto tin sites, and the broader signal located at 2271 cm^–1^ showed an increased intensity. This result suggests that the acidity of tin sites has been weakened by the addition of the KOH base. However, as evidenced from the catalytic activity results, this does not involve the inactivation of the catalytic sites but the depression of the side reactions conducting to polycondensed bulky side products, which finally leads to the deactivation of the catalysts.

**Figure 5:**
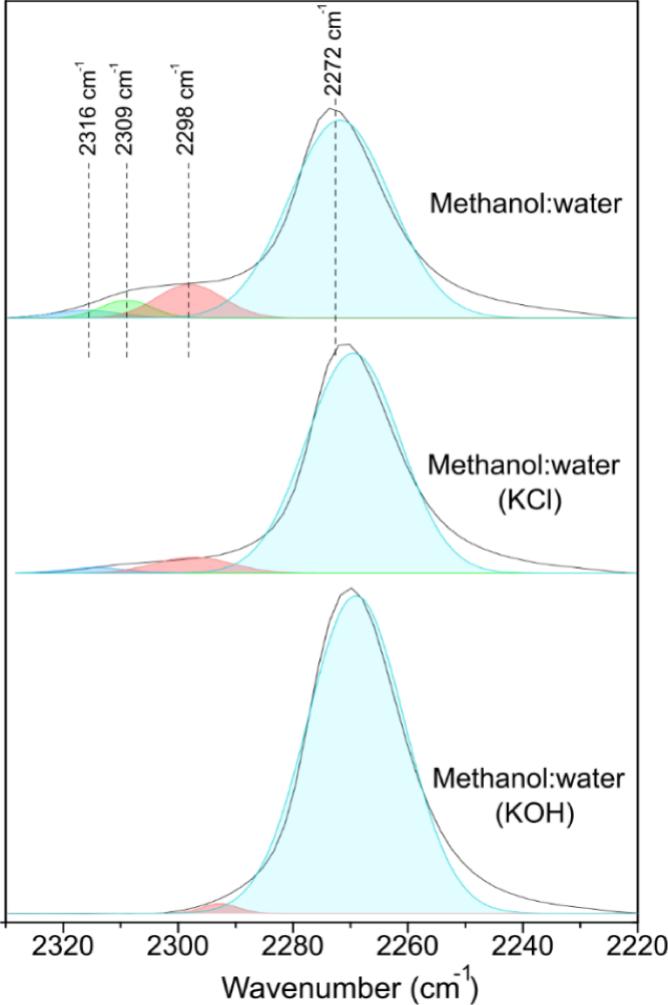
FTIR spectra of deuterated acetonitrile adsorbed on the [K]­Sn-USY catalyst exposed to different reaction media (methanol:water; methanol:water + KCl; methanol:water + KOH).

### Transformation of Complex Sugar Substrates: Hemicellulose Hydrolysates

Having demonstrated the benefits of using small quantities of KOH as a catalyst additive to prevent its deactivation in the treatment of glucose, we have taken a step forward to validate this strategy with a more complex starting feedstock. For this purpose, sugar mixtures corresponding to hemicellulose hydrolysates obtained from Scots pine through an organosolv procedure have been used as starting substrates. Both synthetic sugar mixtures and real hemicellulose hydrolysates have been treated in methanol:H_2_O media with [K]­Sn-USY in the presence of KOH.

#### Emulated Hemicellulose Hydrolysate

An emulated hydrolysate of Scots Pine containing both hexoses and pentoses (glucose, mannose, galactose, xylose, arabinose; Table S2) was selected to evaluate the performance of the [K]­Sn-USY catalyst in a fixed-bed reactor for the transformation of complex sugar mixtures. Similarly to glucose, the treatment of hemicellulose hydrolysate was conducted in the presence of 10 mg/kg of KOH to prevent catalyst deactivation. The analysis of the reactor outstream (Figure 6A) shows a high mass balance, which remains above 80% for 170 h of time-on-stream. Overall, the transformation of the complex sugar mixture emulating Scots pine hemicellulose is conducted with good selectivity, presenting a carbon balance close to 100%. This is a great improvement compared with many studies regarding the transformation of biomass-derived substrates, which usually lack material balance closure. This is a fact that can be ascribed to the degradation of sugars during or before the reaction takes place.^31^ Free sugars, comprising C6—glucose, fructose, mannose, galactose—and C5 sugars—xylose, arabinose, ribose, ribulose, xylulose—are detected in the reaction media, though only mannose and arabinose are present in appreciable concentration, suggesting a lower reactivity of these two monosaccharides, especially when compared to pentoses. The distribution of products is featured, as previously described for individual sugars, by methyl lactate as the main product with 20–25% yield which remains quite stable, with a slight decrease, until the end of operation. The formation of methyl glycosides, MVG, GADMA, and MMHB is also detected though with some differences compared to the transformation of individual sugars. The formation of methyl glycosides from both pentoses and hexoses is still observed; however, there is a higher proportion of the latter, indicating that hexoses are more prone to undergo glycosidation. On the other hand, furanics are produced at very low yield, as expected after the addition of potassium to the reaction media. However, it is evident from the decrease in the sum of product yields derived from retro-aldol cleavage of sugars that the catalyst undergoes a deactivating process, most probably because the causes underlying the formation of organic deposits are still taking place, despite the addition of small amounts of KOH as a catalyst additive.

**Figure 6:**
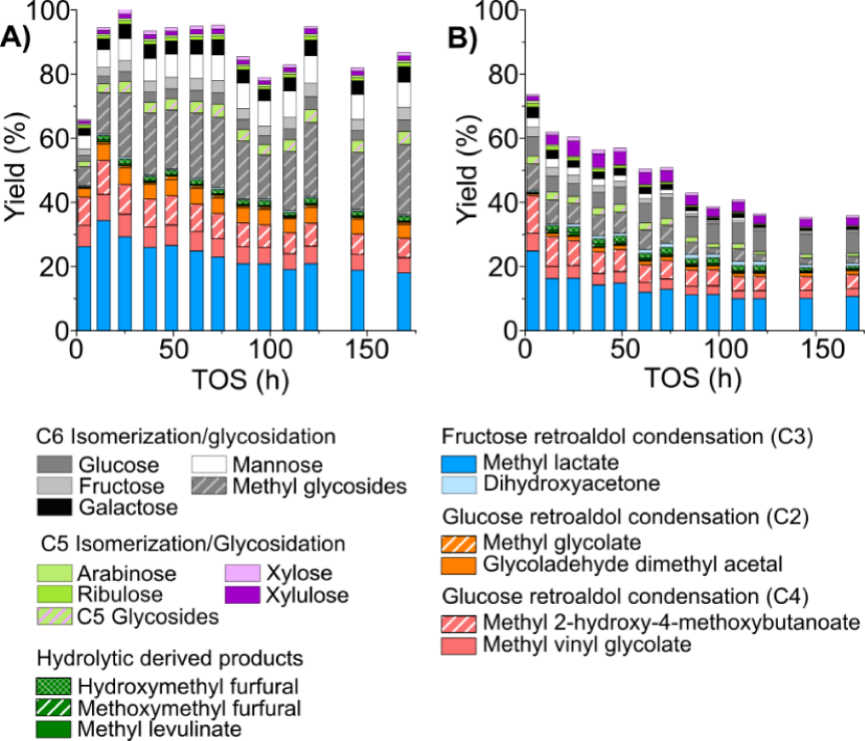
Product distribution obtained in the continuous transformation of Scots Pine hemicellulose (A) emulated and (B) real hydrolysate (B) with [K]­Sn-USY. Conditions: catalyst loading = 0.5 g; reaction medium = methanol:water (96:4 wt:wt); KOH = 10 mg/kg; total sugar concentration = 40 g·L^–1^; WHSV = 0.24 g_sugar_·g_cat_^–1^·h^–1^; reaction temperature = 150 °C; pressure = 13 bar (N_2_ pressurized); N_2_ flow rate = 100 N mL·min^–1^.

#### Real Scots Pine Hemicellulose Hydrolysate

Figure 6B depicts the product distribution obtained in the transformation of a real Scots pine hemicellulose hydrolysate in a fixed bed of the [K]­Sn-USY zeolite. The initial activity exhibited by [K]­Sn-USY in the transformation of the real hydrolysate provides similar yields to the emulated mixture, yielding an equivalent product distribution with similar proportions of MMHB, MVG, and GADMA. However, after the first operation hours and despite a stable product distribution, the general activity of the catalyst presents a sharp decrease, together with increased material balance loss. After this decrease in activity, the yield toward methyl lactate remained stable at values around 15%. The high deactivation rate observed in this case, faster than the deactivation assigned to organic deposition, could be associated with the high amount of C5 and C6 oligosaccharides (see Table S2) in hemicellulose. The proportion of monosaccharides is the same as that used in the emulated substrate, since it was prepared as a mimic of real Scots pine. However, remaining oligosaccharides after hydrolysis of biomass are still present, accounting for 13.3% of the total substrate in the feed stream. These oligosaccharides are difficult to transform, as their previous hydrolysis is required to generate free sugars that undergo retro-aldol cleavage on their way to hydroxyesters. And those oligomers are molecules larger than sugar monosaccharides, which could be a key aspect for the material balance loss, as undesired product formation and oligosaccharide deposition could take place during operation and also deposition over active sites and side product formation. Thermogravimetric and elemental analyses have been performed on spent catalysts after the transformation of both emulated and real Scots pine hemicellulose hydrolysate (see Figure S4C and D). The catalyst used in the transformation of real hemicellulose hydrolysate presents a higher total mass loss, accounting for 16%, indicating a higher level of organic deposition. In addition to that, elemental analysis (see Table S3) indicated a higher carbon content, as well as a higher C/H ratio, which suggests a higher condensation degree of the organic deposits. This is aligned with the presence of large oligosaccharides in the real biomass used as the substrate. However, these results suggest that the [K]­Sn-USY catalyst is a potential candidate for the transformation of hemicellulose hydrolysates after improvement of saccharification of hemicellulose to produce real feed streams, allowing the operation under relevant conditions.

## Conclusions

The [K]­Sn-USY catalyst presents outstanding catalytic activity in the transformation of hexoses and pentoses to produce methyl lactate. However, the use of this catalyst under continuous operation in a fixed-bed reactor presents several challenges associated with the stability of the catalyst. In the transformation of glucose, the composition of the reaction media used as the feed stream contained water as a promoter of catalytic activity; nevertheless, after a long time on stream, the spent catalyst evidence potassium leaching. K lixiviation leads to the formation of Brønsted acid sites, which are responsible for the formation of side products through hydrolyticpathways. These side products—e.g., HMF—tend to form organic deposits, which finally lead to the deactivation of the heterogeneous catalyst. The addition of KCl and KOH to the feed stream as additives was tested to compensate for potassium leaching. KCl partially alleviates the catalyst deactivation by decreasing the deactivation rate. On the other hand, KOH was much more effective in preventing catalyst deactivation due to its better performance in removing Brønsted acidity. This strategy was implemented in the transformation of complex carbohydrate mixtures such as emulated and real hemicellulose hydrolysates. Treating such a complex sugar mixture allowed producing hydroxyesters with quite an outstanding catalyst stability, although some deactivation is still taking place. The complex composition of hemicellulose or the presence of sugar oligomers are the most important causes of catalytic activity decay. These features constitute important challenges to be addressed in the future in the search for a robust and efficient method for hemicellulose valorization.

## Supplementary Material



## Notes

Reaction scheme, experimental details, and additional characterization results (PDF)

## Abbreviations

USY: ultra-stable Y
TOS: time on stream
Py: pyridine
FTIR: Fourier-transformed infrared
DRIFT: diffuse reflectance infrared Fourier transformed
GC: gas chromatography
HPLC: high performance liquid chromatography
C6: hexoses
C5: pentoses
C4: tetroses
C3: trioses
C2: diose
