# Supplementary material for: Continuous Production of Methyl Lactate from Hemicellulosic Sugars: Identifying and Sorting out Sn-USY-Based Catalyst Deactivation
Source: ACS Sustain Chem Eng. 2025 Oct 29;13(44):19088–96. doi: 10.1021/acssuschemeng.5c06986 (PMC12606784; doi:10.1021/acssuschemeng.5c06986)
Supplement: Supplementary file 1 [file sc5c06986_si_001.pdf]

SUPPORTING INFORMATION FOR:

Continuous production of methyl lactate from hemicellulosic sugars: Identifying and sorting out Sn-USY-based catalyst deactivation

José Manuel Jiménez Martín, † Miriam El Tawil Lucas, † Ana Orozco-Saumell, ‡ Manuel López Granados, ‡ José Antonio Pulido, ‡ Rafael Mariscal, ‡ Jovita Moreno, †,※ Alicia García † and Jose Iglesias* †,※*

†*Chemical & Environmental Engineering Group. Universidad Rey Juan Carlos. C/Tulipan s/n, 28933 Madrid, Spain*

‡*Energy and Sustainable Chemistry (EQS) Group. Institute of Catalysis and Petrochemistry, CSIC. C/Marie Curie 2, 28049, Madrid, Spain*

※*Instituto de Tecnologías para la Sostenibilidad. Universidad Rey Juan Carlos. C/Tulipan s/n, 28933. Madrid, Spain*

E-mail: jose.jimenez@urjc.es , jose.iglesias@urjc.es

This document contains 11 pages; 5 figures; 3 tables

Table of content

TABLES

Table S1. Physico-chemical characterization of the prepared [K]Sn-USY catalyst.

Table S2. Composition of hemicellulose hydrolysate recovered from Scots pine.

Table S3. Elemental analysis of spent catalyst after the transformation of glucose, xylose and emulated and real Scots Pine hemicellulose hydrolysate.

SUPPLEMENTARY SCHEMES AND FIGURES

Scheme S1. Reactions pathways undergoing in the transformation of hemicellulose monosaccharaides in methanolic media.

Figure S1. Descriptive scheme of the fixed bed reactor system.

Figure S2. Product distribution obtained in the continuous transformation of glucose with [K]Sn-USY catalyst with intermediate *in situ* calcination. Conditions: catalyst loading = 0.5 g; reaction medium = methanol:water (96:4 wt:wt); feed rate = 0.05 mL·min^{-1}; WHSV = 0.24 $g_{sugar}·g_{cat}^{-1}·h^{-1}$; reaction temperature = 150 ºC; pressure = 13 bar (N_2 pressurized); N_2 flow rate = 100 NmL·min^{-1}.

Figure S3. DRIFT spectra of pyridine adsorbed on USY catalyst at different steps of its synthesis (parent, Sn-functionalized, and K-exchanged).

Figure S4. Thermogravimetric analysis of spent catalyst after the transformation of glucose (A), xylose (B), emulated Scots Pine hydrolysate (C) and real Scots Pine hemicellulose hydrolysate (D).

Figure S5. FTIR spectra collected for [K]Sn-USY fresh catalyst and after continuous transformation of glucose, xylose, emulated and real pine hemicellulose hydrolysate.

TABLES

Table S1. Physico-chemical characterization of the prepared [K]Sn-USY catalyst

Sample	[K]Sn-USY
Al [wt %][a]	0.55
Sn [wt %][a]	1.91
K [wt %][a]	0.40
Acid capacity [meq $H^{+}\cdot g^{-1}$]	0.60
Brønsted/Lewis acid ratio	9.0
S_{BET} [$m^{2}\cdot g^{-1}$]	688
S_{μ} [$m^{2}\cdot g^{-1}$]	459
V_{t} [$cm^{3}\cdot g^{-1}$]	0.45

[a] Measured by ICP-OES.

Table S2. Composition of hemicellulose hydrolysate recovered from Scots pine

Carbohydrates	Concentration (%)
Glucose	15.3
Mannose	30.8
Xylose	19.9
Galactose	9.4
Arabinose	8.1
Oligomers C5	8.1
Oligomers C6	4.2

Table S3. Elemental analysis of spent catalyst after the transformation of glucose, xylose and emulated and real Scots Pine hemicellulose hydrolysate.

	Glucose	Xylose	Emulated Scots Pine	Real Scots Pine
H (wt%)	0.95	0.65	1.04	0.77
C (wt%)	5.21	3.97	6.79	5.48
C/H (wt:wt)	5.49	6.12	6.56	7.09

SUPPLEMENTARY SCHEMES AND FIGURES

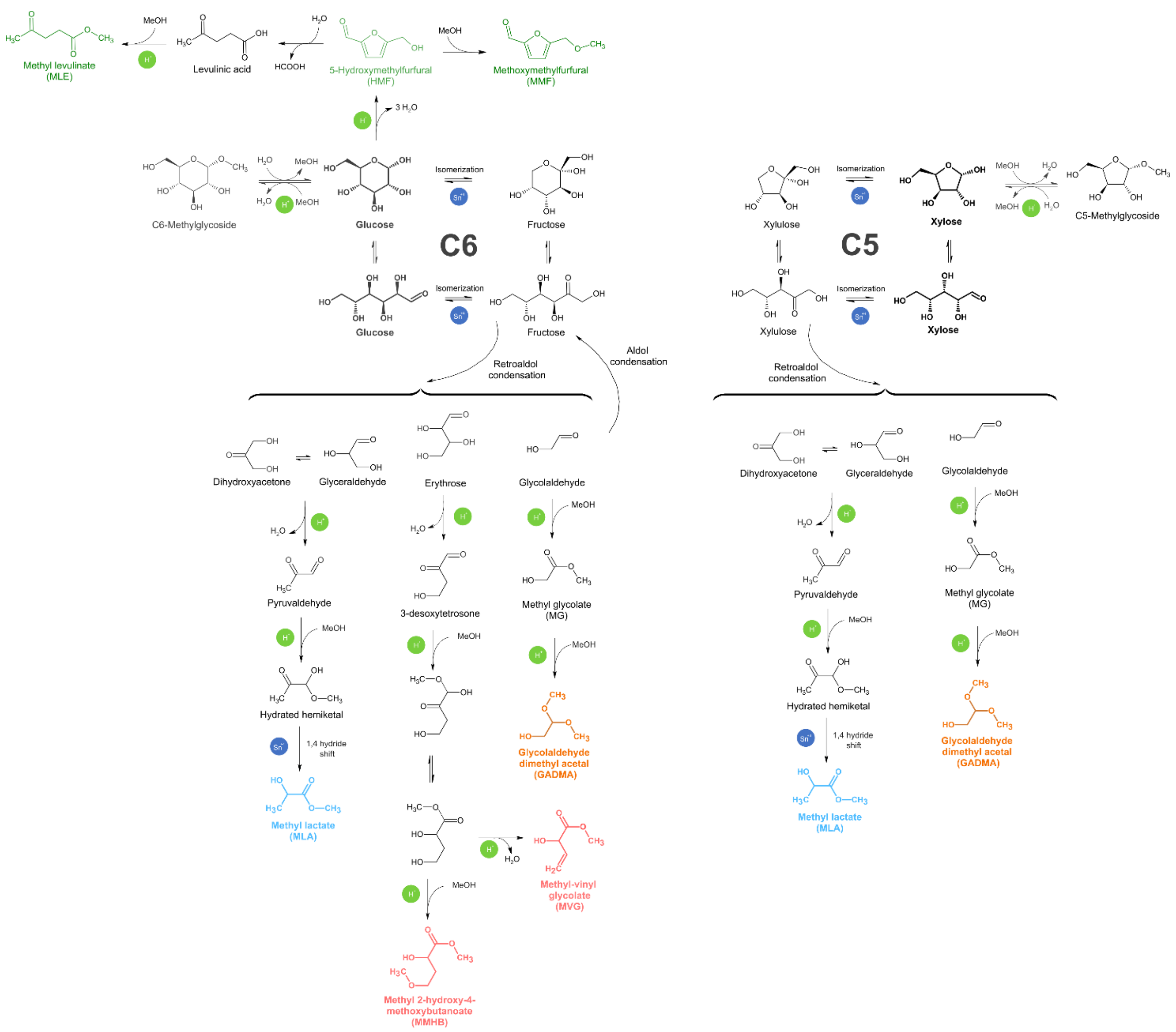

Scheme S1. Reactions pathways undergoing in the transformation of hemicellulose monosaccharaides in methanolic media.

DETAILED DESCRIPTION OF THE REACTION NETWORK

Reactions taking place in the transformation of carbohydrates in the presence of Sn-zeolites involve a complex network of chemical transformations included in Scheme SI-1. In this context, monosaccharides, like glucose or xylose, undergo isomerization to its corresponding ketoses, e.g. fructose, xylulose, in the presence of Sn Lewis acid sites (black products). However, Lewis acid sites can also perform epimerization, conducting to the formation of other aldoses, like mannose or arabinose. All the mentioned sugars can evolve to methyl glycosides in methanol, a transformation catalyzed by Brønsted acid sites, even with low strength (grey products). These glycosides act as sugar reservoirs or masking agents, since their formation is reversible.

Sn Lewis acid sites promote the retro-aldol cleavage of sugar monosaccharides through the adjacent carbon to the carbonyl group. This opens two possibilities in the case of hexoses, the symmetric and asymmetric retro-aldol cleavage of ketohexoses and aldohexoses, respectively. Symmetric retro-aldol splitting of fructose (blue products) produces dihydroxyacetone (DHA) and glyceraldehyde (GLY), which undergo several transformations finally yielding methyl lactate. The asymmetric splitting of the aldohexoses (orange products) leads to the formation of a tetrose and glycolaldehyde (GLA), yielding C4 and C2 products, respectively. On the other hand, for pentoses (maroon products) the splitting always leads to GLA and a C3 fragment (either DHA or GLY), yielding C2 products and methyl lactate (orange and blue products).

C2, C3 and C4 moieties derived from retro-aldol splitting of carbohydrates, can also undergo aldol condensation thus producing 4-, 5- or 6-carbon sugars (even larger, though in minimal quantities) in the presence of Sn Lewis sites. In this way, the combination of aldol condensation and retro-aldol splitting of the sugar substrates makes it possible obtaining a whole collection of different hydroxyacids, ranging from C2 to C4 sugars, under the tested reaction conditions.

Finally, hydrolytic pathways derived products, such as those obtained in the presence of Brønsted acid catalysts can be also obtained (green products). In this type of transformations, a ketose can be dehydrated to form a furanic derivative –5-hydroxymethylfurfural (HMF) or furfural, depending of the carbon number of the starting sugar–. Since the transformation is performed in methanol as reaction medium these products can evolve to the corresponding acetals or, in the case of HMF, to methoxymethyl furfural (MMF) through etherification. This reaction can be catalyzed by Brønsted acid sites.

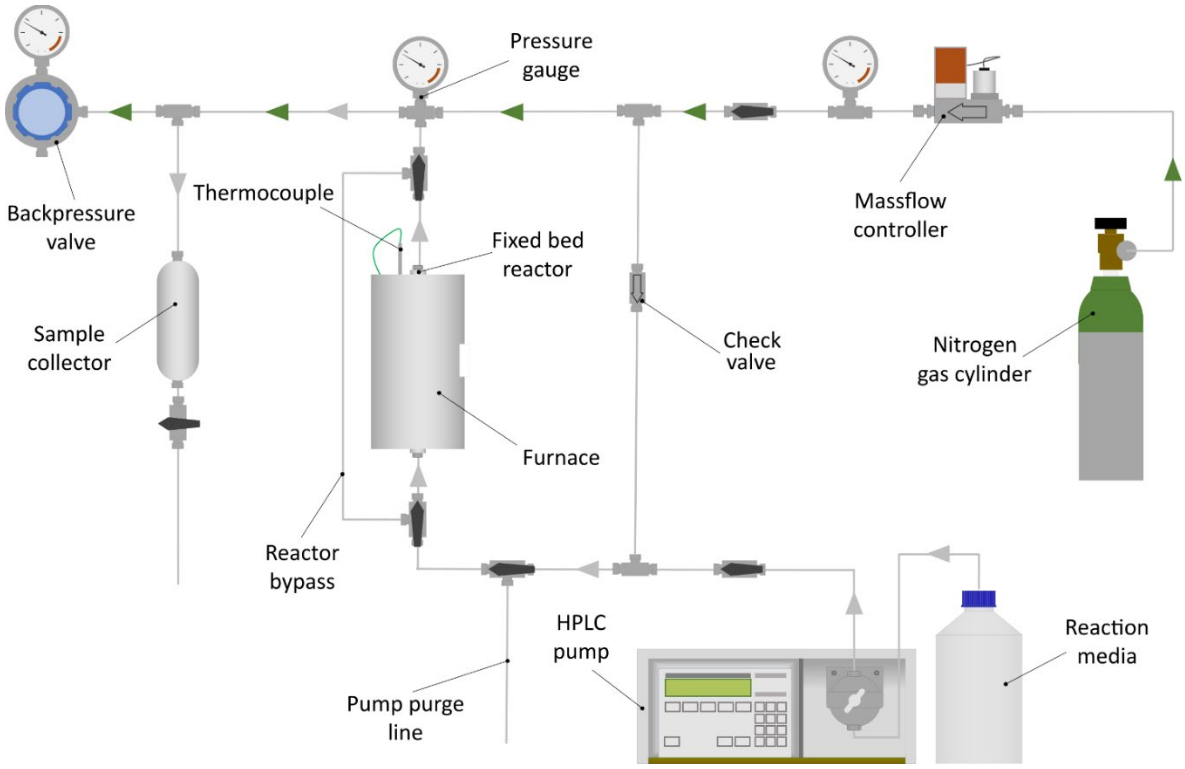

Figure S1. Descriptive scheme of the fixed bed reactor system.

The continuous flow heterogeneous tubular reactor system consists of a tubular fixed-bed reactor with upstream flow, fed by a Gilson 305 piston HPLC pump with liquid feed stream. The whole system is pressurized with nitrogen to allow continuous operation, including the stainless-steel vessel placed at the reactor outstream as a sample collector to collect the samples in liquid state. The pressure in the system is controlled with a backpressure valve placed at the end of the line. The reactor is heated with a shell-built furnace, allowing the tubular reactor assembly and disassembly. The temperature in the reactor is monitored by a K-type thermocouple and controlled by an Osaka QB 48 PID controller.

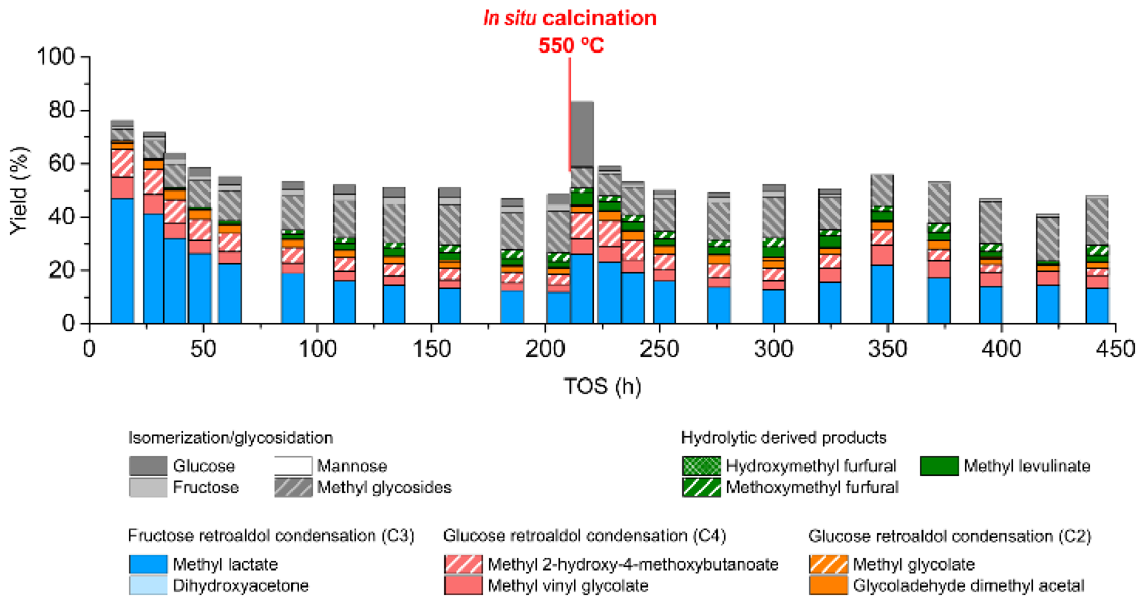

Figure S2. Product distribution obtained in the continuous transformation of glucose with [K]Sn-USY catalyst with intermediate in situ calcination. Conditions: catalyst loading = 0.5 g; reaction medium = methanol:water (96:4 wt:wt); feed rate = 0.05 mL·min-^{1}; WHSV = 0.24 $g_{sugar}\cdot g_{cat}^{-1}\cdot h^{-1}$; reaction temperature = 150 ºC; pressure = 13 bar (N_2 pressurized); N_2 flow rate = 100 NmL·min^{-1}.

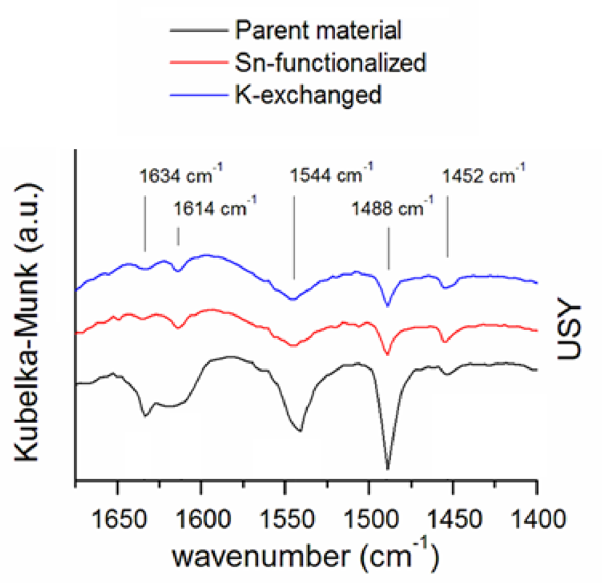

Figure S3. DRIFT spectra of pyridine adsorbed on USY catalyst at different steps of its synthesis (parent, Sn-functionalized, and K-exchanged).

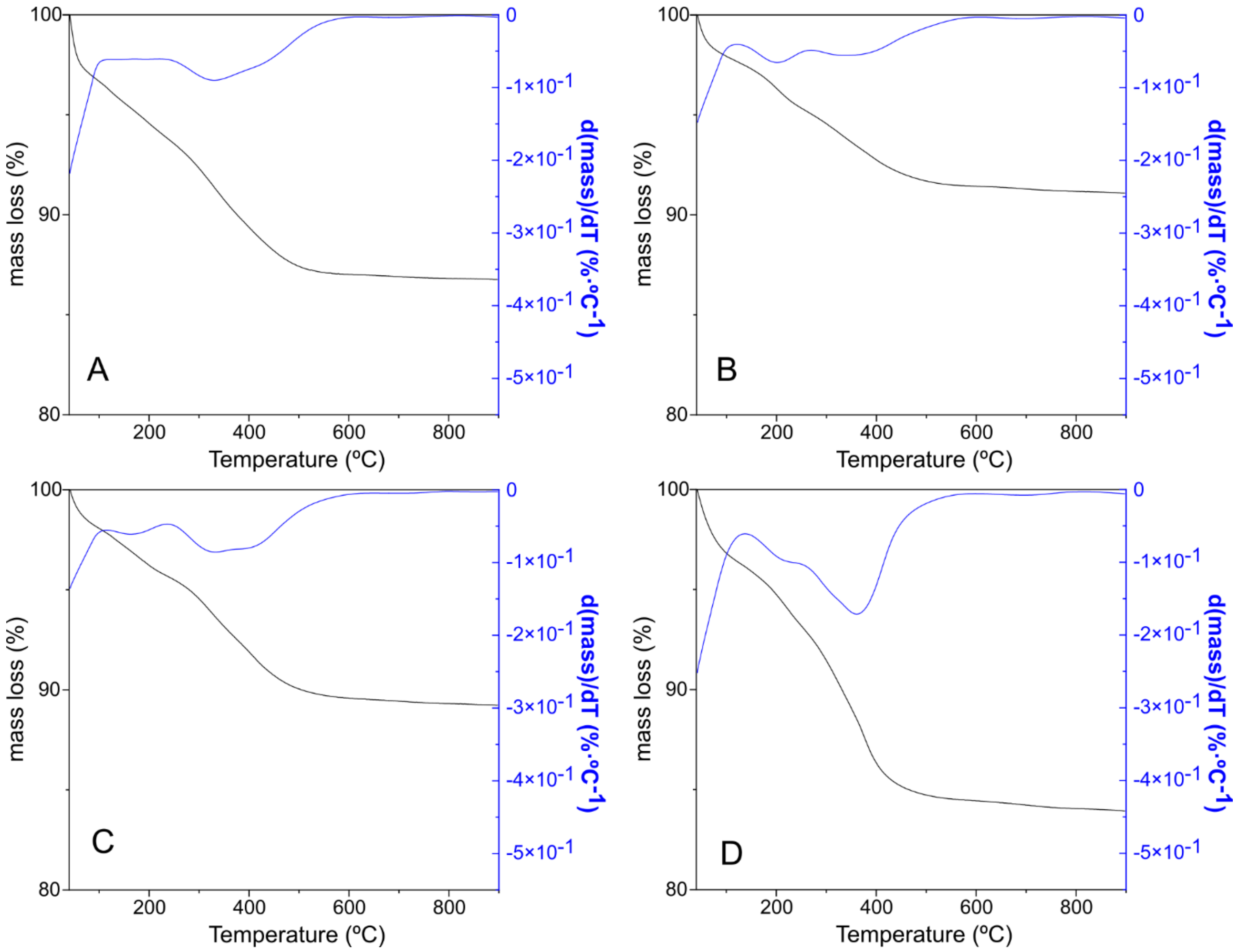

Figure S4. Thermogravimetric analysis of spent catalyst after the transformation of glucose (A), xylose (B), emulated Scots Pine hydrolysate (C) and real Scots Pine hemicellulose hydrolysate (D).

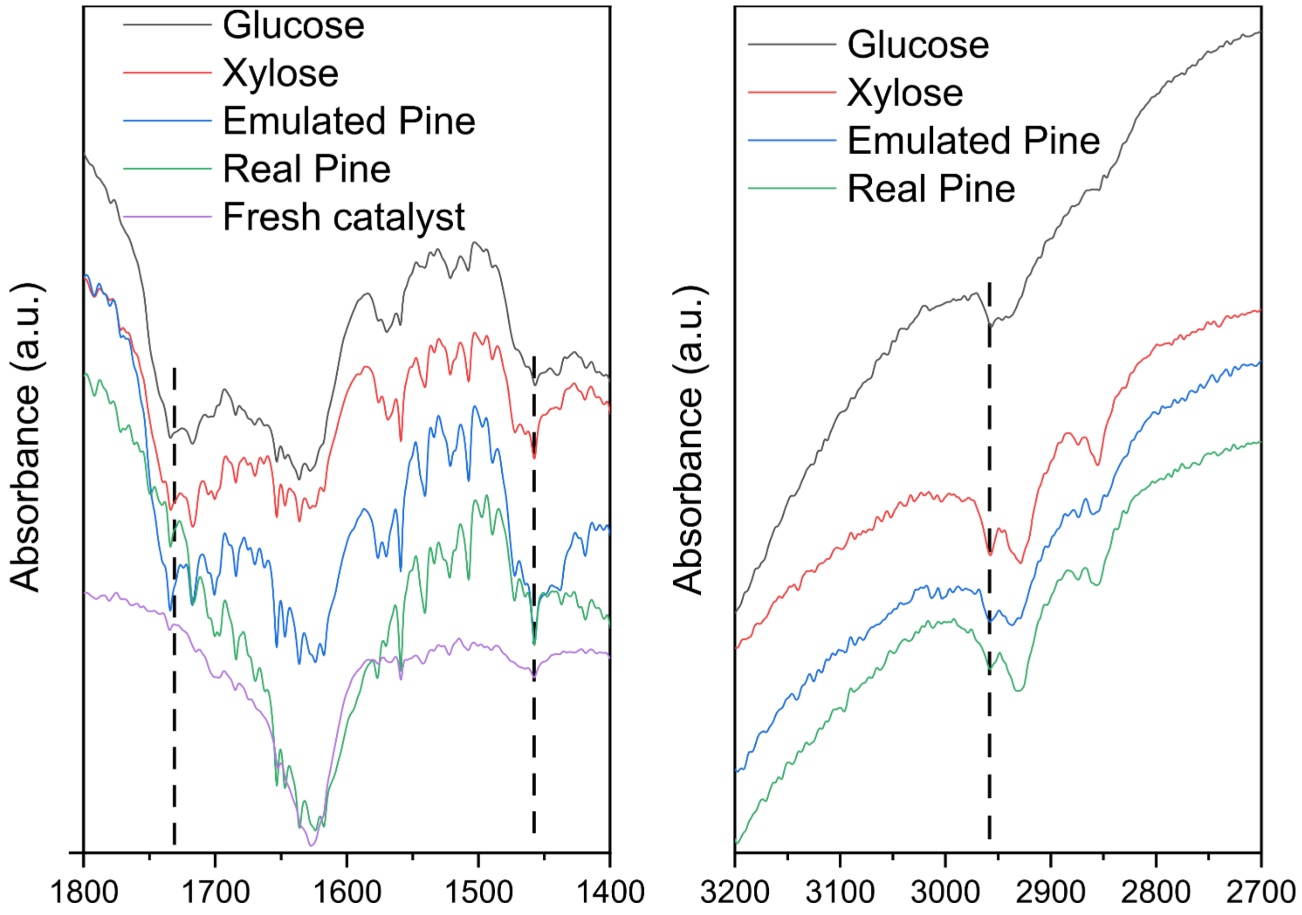

Figure S5. FTIR spectra collected for [K]Sn-USY fresh catalyst and after continuous transformation of glucose, xylose, emulated and real pine hemicellulose hydrolysate.
